# Peripheral Blood miRNA Expression in Patients with Essential Hypertension in the Han Chinese Population in Hefei, China

**DOI:** 10.1007/s10528-024-10867-6

**Published:** 2024-06-21

**Authors:** Bin Cheng, Ronglu Yang, Hui Xu, Li Wang, Nan Jiang, Tingting Song, Changwu Dong

**Affiliations:** 1 Department of Anhui University of Chinese Medicine, Anhui University of Chinese Medicine, Hefei, China; 2 Department of Traditional Chinese Medicine, The First Affiliated Hospital of USTC, Division of Life Sciences and Medicine, University of Science and Technology of China, Hefei, China; 3 College of Traditional Chinese Medicine, Anhui University of Chinese Medicine, Hefei, China; 4 The First Clinical Medical School, Anhui University of Chinese Medicine, Hefei, China; 5 The Second Clinical Medical School, Anhui University of Chinese Medicine, Hefei, China

**Keywords:** microRNAs, Essential hypertension, Plasma

## Abstract

Primary hypertension is a significant risk factor for cardiovascular diseases. However, the pathogenesis of primary hypertension involves multiple biological processes, including the nervous system, circulatory system, endocrine system, and more. Despite extensive research, there is no clear understanding of the regulatory mechanism underlying its pathogenesis. In recent years, miRNAs have gained attention as a regulatory factor capable of modulating the expression of related molecules through gene silencing. Therefore, exploring differentially expressed miRNAs in patients with essential hypertension (EH) may offer a novel approach for future diagnosis and treatment of EH. This study included a total of twenty Han Chinese population samples from Hefei, China. The samples consisted of 10 healthy individuals and 10 patients with EH. Statistical analysis was conducted to analyze the general information of the two-sample groups. High-throughput sequencing and base identification were performed to obtain the original sequencing sequences. These sequences were then annotated using various databases including Rfam, cDNA sequences, species repetitive sequences library, and miRBase database. The number of miRNA species contained in the samples was measured. Next, TPM values were calculated to determine the expression level of each miRNA. The bioinformatics of the differentiated miRNAs were analyzed using the OECloud tool, and RPM values were calculated. Furthermore, the reliability of the expression was analyzed by calculating the area under the Roc curve using the OECloud tools. Statistical analysis revealed no significant differences between the two samples in terms of age distribution, gender composition, smoking history, and alcohol consumption history (*P* > 0.05). However, there was a notable presence of family genetic history and high BMI in the EH population (*P* < 0.05). The sequencing results identified a total of 245 miRNAs, out of which 16 miRNAs exhibited differential expression. Among the highly expressed miRNAs were let-7d-5p, miR-101-3p, miR-122-5p, miR-122b-3p, miR-192-5p, and miR-6722-3p. On the other hand, the lowly expressed miRNAs included miR-103a-3p, miR-16-5p, miR-181a-2-3p, miR-200a-3p, miR-200b-3p, miR-200c-3p, miR-221-3p, miR-30d-5p, miR-342-5p, and miR-543. This study initially identified 16 miRNAs that are aberrantly expressed and function in various processes associated with the onset and progression of essential hypertension. These miRNAs have the potential to be targeted for future diagnosis and treatment of EH. However, further samples are required to provide additional support for this study.

**Supplementary Information:**

The online version contains supplementary material available at 10.1007/s10528-024-10867-6.

## Introduction

As one of the most widespread non-infectious, multifactorial diseases in the world today, essential hypertension (EH) is also an influential risk factor for various cardiovascular and cerebrovascular diseases (Heidari et al. 2022). With the rapid development of medical and biological technologies, the study of genes associated with the development of EH has been a topic of concern. Over the past few decades, great efforts have been made to identify genes and chromosomal loci associated with blood pressure traits or hypertension (Wang et al. 2023a, b). Globally, about 26.4% of adults suffer from varying degrees of hypertension (Zhang et al. 2023). In previous studies, Mendelian genetics and genome-wide screens have identified variant genes associated with hypertension, but these genes collectively explain only 2% to 3% of blood pressure fluctuations (Burrello et al. 2017). This figure, on the contrary, far exceeds the proportion of variants explained by Mendelian inheritance and genome-wide screening, and it is unlikely that such a high incidence is due entirely to genetic mutations. In recent years, developments in epigenetics have given a plausible explanation for the increasing incidence year by year; external factors affect epigenetic alterations in genes associated with hypertension, which in turn cause changes in blood pressure (Arif et al. 2019).

MicroRNAs (miRNAs) are a class of small non-coding RNAs about 21 to 26 nucleotides long that act as post-transcriptional regulators to regulate the expression of endogenous genes and thus affect protein synthesis. Consequently, we hypothesize that a collection consisting of numerous miRNAs is likely to reveal the underlying pathological and physiological processes of the disease.

The pathogenesis of EH is a complex process that involves various genetic and metabolic signaling systems. miRNAs play a significant role in regulating key genes and have important biological functions in vascular remodeling and organ damage, particularly in the heart and kidneys (Wang et al. 2023a, b). It has been reported that miRNAs can serve as novel biomarkers for the pathogenesis of EH and become new targets for the prevention and diagnosis of EH (Jusic et al. 2023). The miR-125a-5p/miR-125b-5p pathway enhances the pathological activation of angiotensin II-AT1R in mouse distal convoluted tubule cells by inhibiting Atrap. Conversely, Ang II, acting through AT1R, controls the expression of miR-138, miR-132, miR-212, and miR-26a, resulting in increased blood pressure (Colpaert et al. 2019; Zhang et al. 2021; Hirota et al. 2023). Notwithstanding, due to the cross-regulatory mechanism between miRNAs and mRNAs, it is extremely difficult for single site miRNA targeting studies to effectively elucidate the molecular level changes in patients during the pathogenesis and progression of EH.

To further explore which miRNAs undergo aberrant expression during the development of EH. We detected miRNA data in peripheral blood samples from EH patients and compared them with normal population. Subsequently, we combined bioinformatics-related analysis methods to study the regulatory network of miRNAs and their target mRNAs in the pathogenesis of EH, with a view to explaining the pathogenesis of EH at the molecular level.

## Material and Methods

### Subjects

Between July and December 2022, a total of 10 EH patients (Case Group) and 10 healthy subjects (Control Group) were enrolled from the Second Affiliated Hospital of Anhui University of Traditional Chinese Medicine. All patients diagnosed with EH were newly diagnosed, and the diagnosis was made according to the following criteria: (1) office systolic blood pressure (SBP) ≥ 140 mmHg and/or diastolic pressure (DBP) ≥ 90 mmHg after multiple repeated measurements; (2) the study population consisted solely of adults (> 18 years of age); and (3) patients with white-coat hypertension, secondary hypertension, gestational hypertension, and other diseases were excluded. The study was conducted in accordance with *the Declaration of Helsinki*, and each patient provided informed consent for participation in the research. The Ethics Committee of the Second Affiliated Hospital of Anhui University of Traditional Chinese Medicine approved the study with the approval number 2022-zj-02.

### Sample Collection

All blood samples were collected after overnight fasting, before 7:00 a.m. Following collection, the samples were promptly stored at a low temperature and centrifuged within 1 h to separate the plasma. The separation process involved centrifugation at 4 °C and a speed of 3000 rpm for 10 min. Once centrifugation was complete, plasma was carefully collected using a pipette gun, dispensed into 1 ml freezing tubes, labeled with the corresponding sample number, and transferred to a − 80 °C refrigerator for storage.

### Small RNA Extraction and Library Construction

Total RNA was extracted from plasma samples using the mirVana miRNA isolation kit (Ambion). Quantitative analysis of total RNA was performed with a Nanodrop 2000 (Thermo Fisher Scientific Inc., USA). RNA integrity was assessed using an Agilent 2100 Bioanalyzer (Agilent Technologies, Inc., USA). For Small RNA library construction, 1 μg of total RNA from each sample was used with the TruSeq Small RNA Sample Preparation Kit (catalog number RS-200-0012, Illumina, USA) following the manufacturer’s recommendations. Briefly, total RNA was ligated to an adapter at each end, and then the adapter-conjugated RNA was reverse-transcribed to cDNA and PCR-amplified. Finally, the 140–160 bp PCR products were isolated and purified as Small RNA libraries. Small RNA sequencing and analysis were performed by OE Biotech Ltd Co. (Shanghai, China).

### Sequence Comparison Annotation

Base reads are converted to sequence data, also known as raw data/reads, through base calling. Low quality reads are filtered out, and reads containing 5′ primer contaminants and poly (A) are removed. Clean reads are obtained by filtering out reads without 3′ adapter and insert tags, as well as reads shorter than 15 nt or longer than 41 nt in the raw data. The length distribution of clean sequences in the reference genome was determined for preliminary analysis. Non-coding RNAs, such as rRNA, tRNA, small nuclear RNA (snRNA), and others, were annotated. These RNAs were aligned and subjected to a Bowtie (Langmead et al. 2009) search using Rfam v.10.1 (http://www.sanger.ac.uk/software/Rfam) (Griffiths-Jones et al. 2003). Known miRNAs were identified by comparing them with the miRBase v22 database (http://www.mirbase.org/) (Griffiths-Jones et al. 2008), and the expression patterns of the known miRNAs in different samples were analyzed. Unannotated reads were then analyzed with mirdeep2 to predict new miRNAs (Friedländer et al. 2012). The corresponding miRNA star sequence and miRNA mature sequence were also identified based on the hairpin structure of the former miRNA and the miRBase database.

### miRNA Expression Analysis

The abundance of a miRNA directly reflects its expression level, with higher miRNA abundance indicating higher expression. To determine the expression level of miRNAs in small RNA sequencing analysis, we counted the sequences localized to the mature body of the species and the newly predicted miRNA sequences. We performed expression counts for both known and newly predicted miRNAs. The miRNA expression was calculated using the TPM (transcript per million) computational metric, which is calculated as TPM = N/M × 10^6^. Here, N represents the number of reads compared to each miRNA, and M represents the total number of compared reads in the sample (Clean). We determined miRNA expression levels by localizing them to the mature body sequences of the species and counting the newly predicted miRNA sequences in the small RNA sequencing analysis. We presented the results of miRNA expression using box-and-line plots, and also plotted the density of TPM values as a distribution curve to reflect the miRNA expression pattern of each sample.

### miRNA Differential Analysis

Differentially expressed miRNAs were calculated and screened with a *p*-value less than 0.05 as a threshold. The *p*-value was calculated using the DEG algorithm (Burden et al. 2014) in the R package for experiments with biological replicates and the Audic-Claverie statistic (Tino 2009) for experiments without biological replicates.

We performed online analysis of miRNA dataset expression differences between EH and normal groups by the OECloud tools (https://cloud.oebiotech.com) and developed data screening rules: the *p*-value < 0.05 and |log 2FC|> 1, and obtained differentially expressed miRNAs (DEs). In order to show the differential expression of miRNAs and mRNAs in a variety of samples, we draw a volcano plot and a heat map.

### DEs Target Gene Prediction and Function Analysis

The targets of differentially expressed miRNAs were predicted using software miranda (Enright et al. 2003) in animal, with the parameter as follows: S ≥ 150, ΔG ≤  − 30 kcal/mol and demand strict 5′ seed pairing. GO enrichment and KEGG pathway enrichment analysis of different expressed miRNA-target-Gene were respectively performed using R based on the hypergeometric distribution.

### ROC Curves

ROC curves (Martínez et al. 2023) were constructed using Prism 9.0 software (GraphPad Software, Inc.) to differentiate EH patients from controls based on plasma miRNAs. The area under the ROC curve (AUC) was analyzed to evaluate the diagnostic accuracy of each identified miRNA.

### Statistical Analysis

In the general data, we statistically analyzed the gender, age, history of smoking, history of alcohol consumption, family genetic history, blood pressure values and BMI, where the two-sample independent *t*-test was used for measurement data, and the Fisher’s exact test was used for count data, with *p* < 0.05 as the test criterion. Statistical analysis was performed using SPSS 22.0 statistical software (SPSS, Chicago).

## Results

### General Information

Based on the statistical results presented in Table 1, no significant differences were observed between the Case and Control groups in terms of age distribution, gender composition, smoking history, and drinking history. However, it is worth noting that the Case group exhibited a significant family genetic history, and there was also a notable increase in body mass index among the EH population compared to the normal population.

**Table 1:** Statistical table of basic information of samples

Characteristic	Group	Case group *N* = 10	Control group *N* = 10	*p*
Age (years)	–	63.900 ± 8.062	63.800 ± 8.364	0.979
Gender	Male	6	5	1.000
	Female	4	5	
Smoking	Yes	6	2	0.170
	None	4	8	
Alcohol consumption	Yes	4	5	1.000
	None	6	5	
Family history	Yes	7	1	0.020^*^
	None	3	9	
SBP (mmHg)	–	155.300 ± 9.093	130.400 ± 6.150	0.000^*^
DBP (mmHg)	–	85.700 ± 13.090	74.200 ± 4.686	0.018^*^
BMI	–	25.191 ± 4.242	21.064 ± 1.667	0.010^*^

*p < 0.05

### Small RNA Sequencing Data Preprocessing

**Fig. 1:**
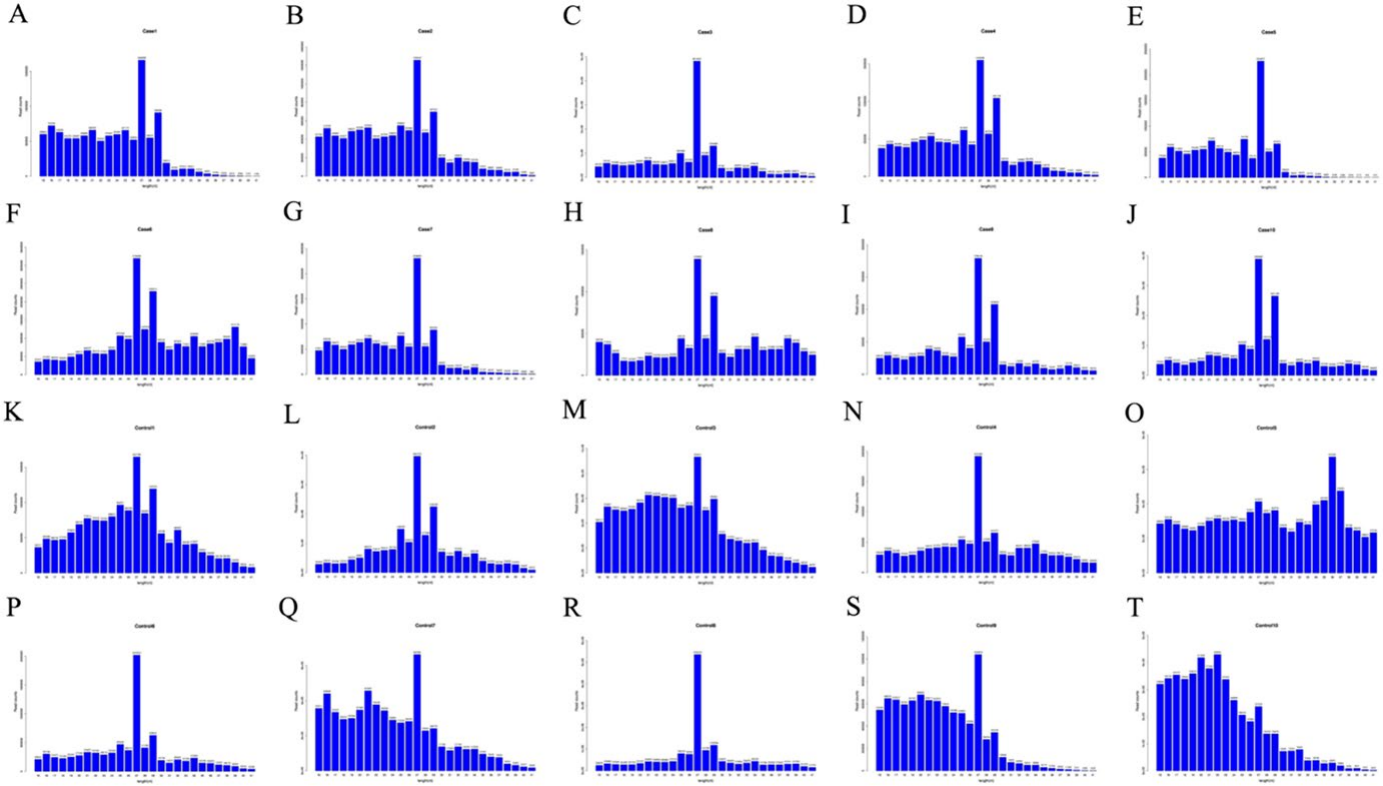
Clean reads length distribution bar chart. **A**–**J** Case 1–10; **K**–**T** Control 1–10

After removing primer and junction sequences, and conducting quality check and length screening of sequencing fragment bases, reliable quality sequencing fragments were selected. Subsequently, the species (denoted by Unique) and quantity (denoted by Total) of small RNAs were counted, and the length distribution statistics of miRNAs were performed (Fig. 1). Generally, small RNAs have a length interval of 18–30 nt, and the peak of the length distribution can aid in determining the species of small RNA. For instance, miRNAs typically have a length concentration of 21–25 nt, siRNAs have a length concentration of 20–25 nt, and piRNAs have a length concentration of 30 nt.

### Statistics of Known miRNA Species Detected in Each Sample

**Fig. 2:**
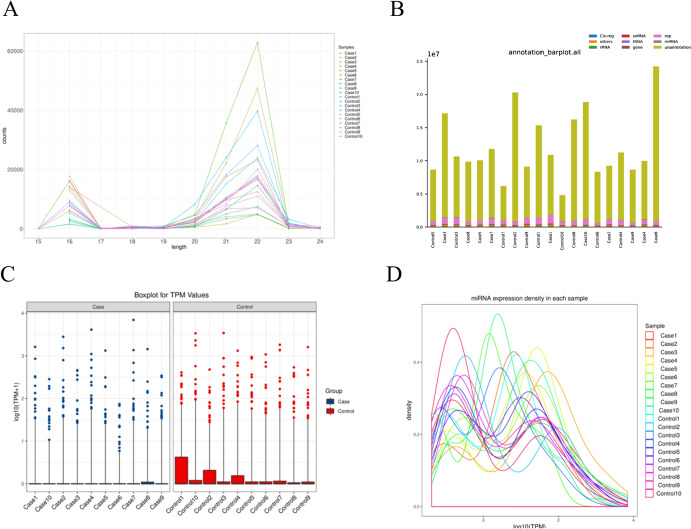
miRNA species and expression in samples. **A** Known line plot of miRNA length distribution. **B** Categorical annotation histogram. **C** Sample expression box plot. **B** TPM density distribution chart

**Fig.3:**
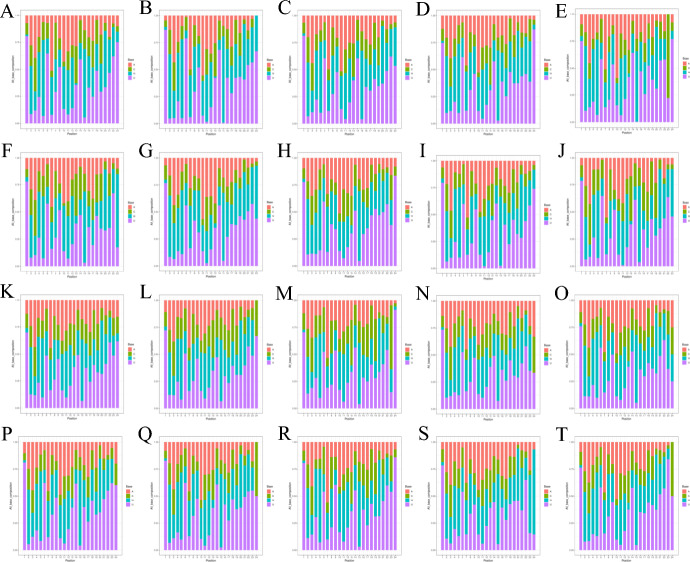
Statistical chart of each base preference. **A**–**J** Case 1–10; **K**–**T** Control 1–10; Red represents adenine, green represents cytosine, blue represents guanine, and purple represents uracil

The preprocessed assay data were annotated sequentially with the Rfam database, cDNA sequences, species repetitive sequence libraries, and miRBase database. The distribution of small RNAs in the samples was then counted, and the results are presented in Table 2 and Fig. 2B. Among them, Table 3 and Fig. 2A display the number of known miRNA species detected in each sample. Additionally, the miRNA base preferences are illustrated in Fig. 3. By calculating the TPM values of the known miRNAs detected in each sample, we obtained the relative expression of 245 miRNAs, and the results of the box-and-line plot are shown in Fig. 2C. The density distribution curve of TPM values is shown in Fig. 2D.

**Table 2:** Total classification comments of each sample reads

Annotation type	Number of total	% of total (%)	Number of uniq	% of uniq (%)
rRNA	29,842	0.22	476	0.19
tRNA	3914	0.03	83	0.03
snRNA	4650	0.03	268	0.11
Cis-reg	18,537	0.14	326	0.13
Other Rfam RNA	9076	0.07	603	0.24
gene	12,660	0.09	1808	0.71
Repeat	7,070,292	52.41	28,798	11.34
Known miRNA	38,092	0.28	627	0.25
Unannotation	6,303,185	46.72	220,899	87.01

**Table 3:** Statistics on known miRNA detection rates

Sample	Known miRNA categories	Sample	Known miRNA categories	Sample	Known miRNA categories	Sample	Known miRNA categories
Case 1	45	Case 6	46	Control 1	102	Control 6	67
Case 2	28	Case 7	42	Control 2	99	Control 7	67
Case 3	52	Case 8	66	Control 3	78	Control 8	67
Case 4	55	Case 9	38	Control 4	88	Control 9	76
Case 5	33	Case 10	39	Control 5	80	Control 10	67

### Differential miRNA Expression Analysis Between Two Groups of Samples

**Fig. 4:**
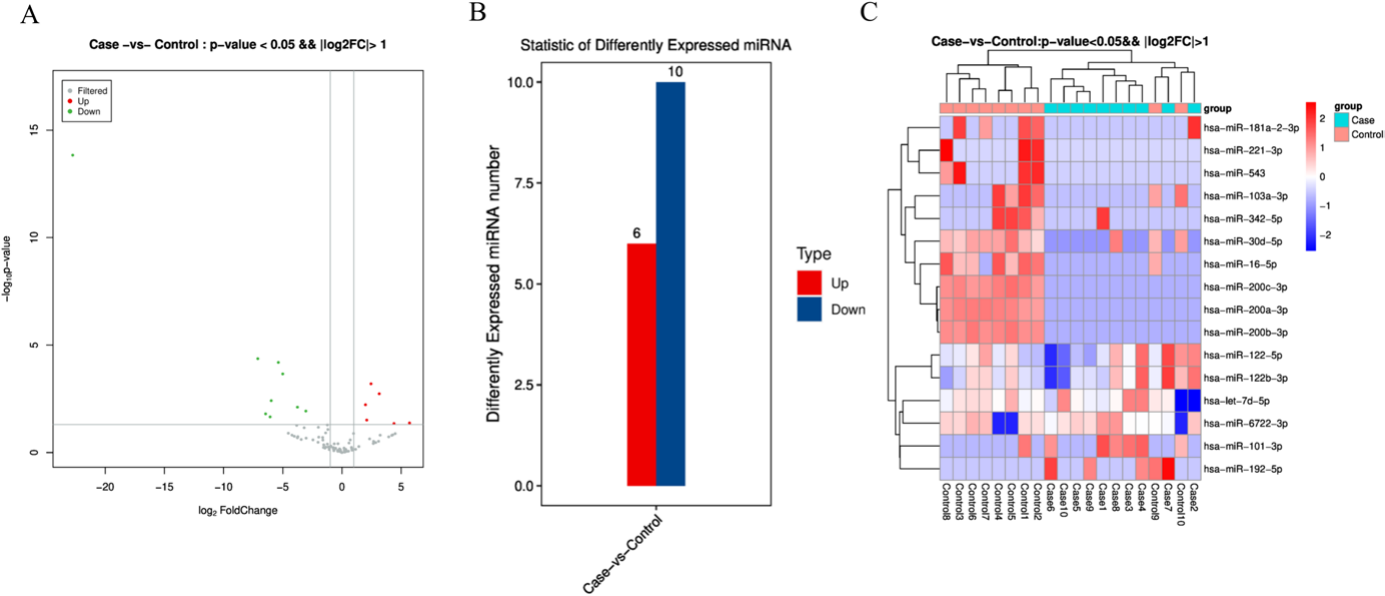
Analysis of differentially expressed miRNAs between case and control group. **A** Volcano map of DEs. **B** Statistical barplot of differential miRNAs. **C** Heat map of Des

Based on the screening criteria, we identified 16 dysregulated miRNAs (Table 4), including 10 down-regulated miRNAs and 6 up-regulated miRNAs (Fig. 4B). To visually illustrate the distribution of miRNA expression differences between the Case and Control blocking groups, we created a volcano plot with “− log10 (*P*. Value)” as the vertical coordinate and “log FC” as the horizontal coordinate (Fig. 4A). Furthermore, we generated a heat map to display the differences between the two groups (Fig. 4C).

**Table 4:** 16miRNA in DEs

Symbol	Up/down	LogFC	*P*-value
let-7d-5p	Up	3.143	0.002
miR-101-3p	Up	4.391	0.045
miR-122-5p	Up	1.983	0.006
miR-122b-3p	Up	2.445	0.001
miR-192-5p	Up	5.701	0.042
miR-6722-3p	Up	2.091	0.031
miR-103a-3p	Down	− 5.978	0.004
miR-16-5p	Down	− 7.114	0.000
miR-181a-2-3p	Down	− 6.451	0.016
miR-200a-3p	Down	− 5.377	0.000
miR-200b-3p	Down	− 5.009	0.000
miR-200c-3p	Down	− 3.765	0.008
miR-221-3p	Down	− 22.750	0.000
miR-30d-5p	Down	− 3.057	0.012
miR-342-5p	Down	− 6.075	0.022
miR-543	Down	− 23.253	0.000

### Enrichment Analysis of Target Genes for DEs

**Fig. 5:**
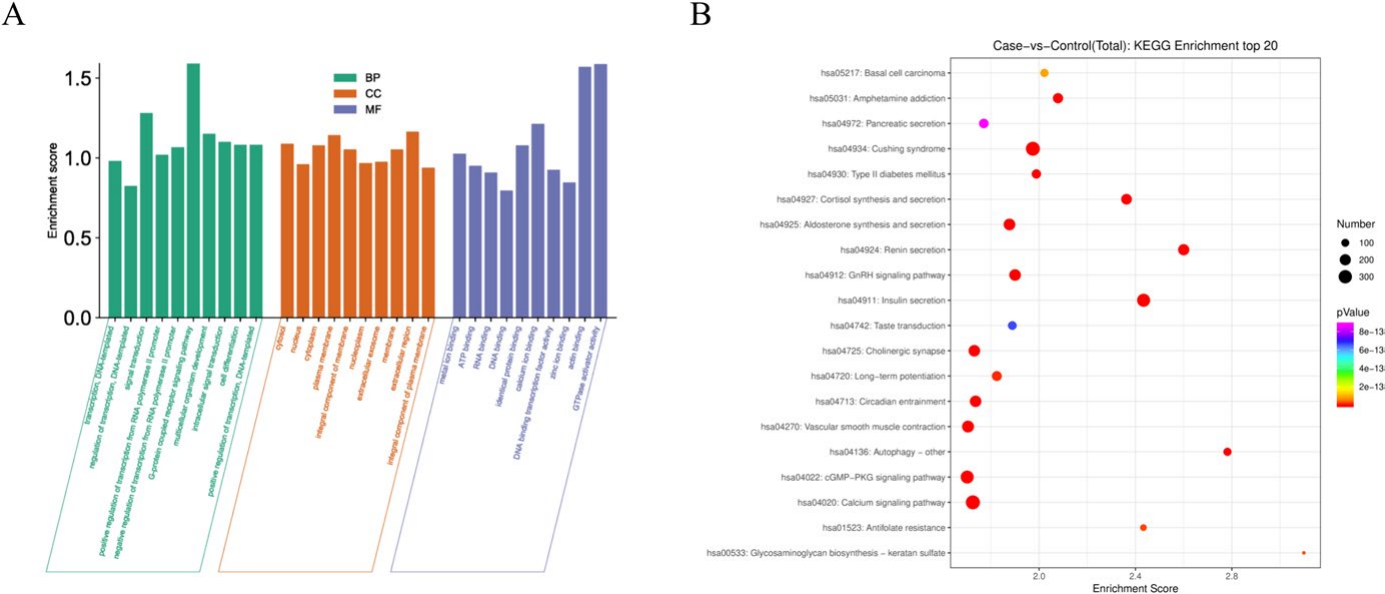
Top 10 significant enrichment GO and Top 20 significant enrichment KEGG terms of DEs. **A** GO: biological process (BP), cellular component (CC) and molecular function (MF). **B** KEGG: signaling pathway

To investigate the function of miRNA target genes, we predicted DEs target genes using the miranda software (refer to Supplementary Material for detailed results) and conducted GO annotation and KEGG pathway analysis using R software. Figure 5 presents the top 10 significantly enriched BPs, CCs, MFs, and KEGG pathways. The BPs enrichment analysis revealed that the more significant results were related to G-protein coupled receptor signaling pathway, signal transduction, and multicellular organism development. The top three CCs included Extracellular region, Plasma membrane, and Cytosol. The most enriched MFs were GTPase activator activity, Actin binding, and Calcium ion binding. In the KEGG pathway analysis, signaling pathways associated with hypertension, such as Renin secretion, Calcium signaling pathway, Aldosterone synthesis and secretion, and Vascular smooth muscle contraction, were enriched.

### ROC Curves for Each Sample

**Fig. 6:**
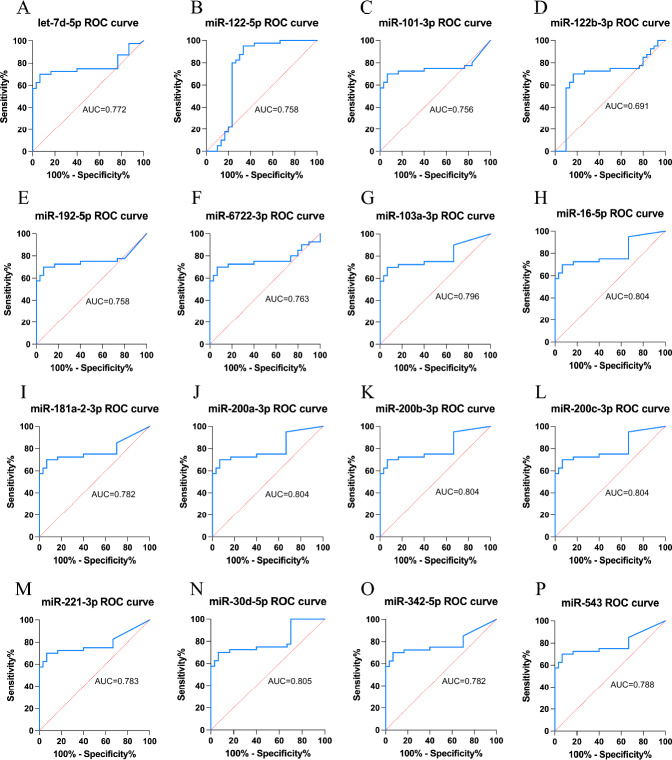
DEs ROC graph. **A**–**F** Up-regulated miRNAs. **G**–**P** Down-regulated miRNAs

The ROC curves of Des were analyzed in 20 samples, and the results are displayed in Fig. 6. Notably, miRNAs such as the miR-200 family (AUC = 0.804), miR-16-5p (AUC = 0.804), and miR-30d-5p (AUC = 0.805) exhibited AUC values greater than 0.8.

## Discussion

The causes of primary hypertension are primarily believed to be associated with environmental and genetic factors. These factors work in conjunction to induce varying degrees of sympathetic hyperfunction (Seravalle et al. 2022), abnormal expression of the renin–angiotensin–aldosterone system (RAAS) (Mohamed et al. 2022), sodium retention (Suzumoto et al. 2023), impaired vascular endothelial cell function (de Oliveira et al. 2022), insulin resistance (Sinha et al. 2022), and immune mechanisms (Drummond et al. 2019), among other microscopic reactions within the human body. However, a number of substances are involved in these complex regulatory processes, the regulation of which has not yet been clearly indicated.

miRNA, a type of small RNA that acts on mRNA, has gained increased attention in recent years due to advancements in epigenetic research. It has become evident that miRNAs play a crucial role in the complex pathogenesis of hypertension by regulating the expression of related molecules and activating signaling pathways (Xu et al. 2023). This study aims to sequence and screen miRNAs in peripheral blood samples from patients with essential hypertension (EH). The objective is to identify abnormally expressed miRNA profiles in EH patients, laying the groundwork for future research on specific regulatory mechanisms.

Based on the findings of this study, it was observed that 6 miRNAs were highly expressed, while 10 miRNAs were found to have low expression levels. In terms of functionality, it was noted that the highly expressed miRNAs are primarily associated with elevated blood pressure and the occurrence of related clinical symptoms. For instance, Let-7d-5p, which is the first miRNA to be confirmed as highly expressed in the human brain, plays a crucial role in regulating various processes such as neuronal differentiation, maturation, degeneration, and regeneration. Furthermore, it is believed to have an impact on the development of psychiatric disorders in humans (Roush et al. 2008). Yang conducted a study to investigate the expression of let-7d-5p in peripheral blood samples from patients with hypertension combined with obstructive sleep apnea-hypoventilation syndrome (Yang et al. 2018). They found that let-7d-5p was highly expressed in these patients, suggesting that it may be a regulatory miRNA involved in causing symptoms such as dizziness, headache, intracranial hypoxia, somnolence, and other psychiatric states in patients with hypertension. The results of the present experiment further support the previous findings, confirming the high expression of let-7d-5p in patients with EH.

miR-101-3p, a widely present miRNA in the human body, has been implicated in the development of various diseases including lung cancer (Dong et al. 2023), diabetes mellitus (Song et al. 2022a, b), and acute gouty arthritis (Shao et al. 2023). In a study investigating vascular growth factor expression in blood samples of depressed adolescents, miR-101-3p was found to be significantly upregulated in depressed patients (Krivosova et al. 2023). Moreover, its target vascular growth factor also showed high expression levels. Previous research has already linked this particular miRNA to psychiatric regulation in humans (Li et al. 2021a, b). Based on these findings, we hypothesized that the high expression of Let-7d-5p and miR-101-3p in EH patients may contribute to increased intravascular pressure and play a role in the regulation of mental status. Consequently, this could lead to the development of unhealthy psychological states, such as anxiety and depression, within the EH population.

In the development of hypertension, certain highly expressed miRNAs play a crucial role in regulating the expression of important proteins. These miRNAs can directly or indirectly contribute to elevated blood pressure. For instance, miR-122-5p has been found to decrease the levels of apelin, elabela, ACE2, and GDF15, while increasing the expression of porimin and CTGF through the regulation of the elabela/apelin-ACE2-GDF15 signaling pathway. This dysregulation leads to significant myocardial fibrosis, inflammation, tumors, and oxidative damage in rats (Song et al. 2022a, b). miR-122b-3p has been found to enhance cell proliferation, apoptosis, and senescence in the body (Gao et al. 2023). On the other hand, upregulation of miR-6722-3p has been shown to significantly decrease the expression of target genes involved in angiogenesis and vascular growth factors, such as STAT3 and IGF-1 (Uchino et al. 2018). Among these target genes, STAT3 plays a crucial role in angiogenesis and extracellular matrix remodeling (Jia et al. 2022). Additionally, IGF-1 promotes the production of nitric oxide by endothelial and vascular smooth muscle cells, thereby safeguarding vascular function (Higashi et al. 2019).

Previous studies have shown that miR-192-5p can prevent hypertension by inhibiting the expression of Atp1b1 (Baker et al. 2019). Additionally, high expression of miR-192-5p has been found to inhibit microvascular endothelial cell proliferation, migration, and angiogenesis, thereby playing a protective role in small blood vessels (Fu et al. 2023). In our current study, we observed high expression of miR-192-5p in the blood of patients with EH, suggesting that this may be a regulatory mechanism within the body.

Low-expressed miRNAs primarily contribute to the protective effects of target organs. They achieve this by either inhibiting the expression of related proteins or by acting on vascular smooth muscle, which helps prevent elevated blood pressure. For instance, miR-181a-2-3p plays a role in inhibiting autophagy in the myocardium by targeting AMBRA1, thereby exerting a protective effect on cardiomyocytes (Li et al. 2022).

Overexpression of miR-221-3p inhibits the protein expression of IGF1R, IGF-2, VEGFR2, and Ang-2 under hypoxic conditions, which reduces the involvement of these molecules in the process of hypertension (Li et al. 2021a, b). miR-543 has been found to be associated with insulin resistance (Hu et al. 2015). Abnormal expression of miR-103a-3p has been observed in pulmonary hypertension and gestational hypertension (Hromadnikova et al. 2015; He et al. 2022). miR-342-5p inhibits cardiomyocyte apoptosis by targeting Caspase 9 and Jnk2, thereby inhibiting hypoxia/reoxygenation-induced cardiomyocyte apoptosis and establishing an endogenous cardioprotective mechanism (Hou et al. 2019). It also promotes the transition of vascular smooth muscle cells (vSMC) from a contractile phenotype to a proliferative and secretory phenotype, which plays a role in regulating blood pressure stabilization (Wen et al. 2023). Low expression of miR-30d-5p fails to attenuate platelet-derived growth factor-induced toxicity in pulmonary artery vascular smooth muscle cells, leading to the development of pulmonary hypertension (Hu et al. 2022). miR-16-5p is an important regulator that promotes phenotypic switching of venous smooth muscle cells, cell phenotypic transition, and its overexpression can inhibit the proliferation and development of venous intima, thus playing a crucial role in lowering blood pressure (Zhang et al. 2022). The miR-200 family, including miR-200a-3p, miR-200b-3p, and miR-200c-3p, is involved in diverse regulatory functions (Magenta et al. 2017). In the cardiovascular field, their high expression can inhibit the proliferation and migration of vascular smooth muscle cells (Du et al. 2022).

The present study investigated 16 miRNAs that are abnormally expressed in patients with essential hypertension. However, there are certain limitations, such as the relatively small sample size, which may introduce bias in the experimental results. Therefore, in future research, the group plans to increase the sample size and conduct larger-scale studies on the abnormally expressed miRNAs. Additionally, the study will focus on longitudinal analysis of individual miRNAs and design experiments to elucidate the specific processes by which miRNAs are involved in essential hypertension.

## Conclusion

This paper investigates the miRNAs present in the peripheral blood of patients with EH. The study initially identifies 16 miRNAs that are expressed abnormally and play a role in different processes related to the occurrence and development of essential hypertension. These miRNAs have the potential to be targeted for future diagnosis and treatment of EH. However, further samples are required to provide additional support for this study.

## Supplementary Information

Below is the link to the electronic supplementary material.

Supplementary file1 (XLSX 11 kb)

Supplementary file2 (XLSX 1890 kb)
